# Older Adults at Risk for Alzheimer’s Disease with Lower Cognitive Scores Exhibit Poorer Skeletal Muscle Health

**DOI:** 10.1093/geroni/igaf122.2415

**Published:** 2025-12-31

**Authors:** Ray Urbina, Michelle Gray, Anthony Campitelli, Megan Jones, Sally Paulson, Joshua Gills, Jordan Glenn

**Affiliations:** University of Arkansas, Fayetteville, Arkansas, United States; University of Arkansas, Fayetteville, Arkansas, United States; College of Idaho, Caldwell, Idaho, United States; University of Arkansas, Fayetteville, Arkansas, United States; University of Houston, Houston, Texas, United States; New York University, New York, New York, United States; SuppCo, Natick, Massachusetts, United States

## Abstract

Alzheimer’s disease (AD) is a major health concern, with early pathology often undetected due to costly assessments. Skeletal muscle health (SMH) deficiencies are strongly linked to AD progression and concurrent declines in physical and cognitive function, suggesting skeletal muscle health as an early biomarker for cognitive decline. This study examines SMH differences between low cognition (LC) and high cognition (HC) in older adults who are at risk for AD. SMH was assessed through physical function tests (PF) – handgrip strength (HG), 5-time sit-to-stand, gait assessments, 6-minute walk, and lower body power (TENDO) – and body composition (DXA). Cognitive health was evaluated using the Repeatable Battery for the Assessment of Neuropsychological Status. Individuals were grouped into low cognition (LC) or high cognition (HC) groups. Independent t-tests were used to analyze differences in means between LC and HC (α = .05). LC consisted of 34 adults (M = 14, F = 20) and HC had 29 adults (M = 6, F = 23). LC had lower performances and poorer outcomes in 16 out of 20 measurements, with significant differences in 10m habitual walk (p=.02) and Handgrip (p=.02). Among females, seven measurements had significant differences between groups – weight (p=.04), lean mass (p=.03), chair stands (p=.02), 10m habitual (p=.02), 10m fast (p=.001), 10m DT habitual (p=.004), and peak power (p=.02). Results show that the LC group demonstrated overall poorer performance and outcomes. These findings align with current literature reinforcing that LC individuals exhibit poorer skeletal muscle health when looking into function which may suggest an increased risk for cognitive decline.

